# Supplementary material for: Metabolic energy sensing by mammalian CLC anion/proton exchangers
Source: EMBO Rep. 2020 May 10;21(6):e47872. doi: 10.15252/embr.201947872 (PMC7271328; doi:10.15252/embr.201947872)
Supplement: Supplementary file 1 — Appendix [file EMBR-21-e47872-s001.docx]

**Apendix**

**Metabolic energy sensing by mammalian CLC anion/proton exchangers**

**Matthias Grieschat^1‡^, Raul E. Guzman^2‡^, Katharina Langschwager^1‡^, Christoph Fahlke^2^, and Alexi K. Alekov^1*^**

^1^Institut für Neurophysiologie, Medizinische Hochschule Hannover, 30625 Hannover, Germany

^2^Institute of Complex Systems, Zelluläre Biophysik (ICS-4), Forschungszentrum Jülich, Leo-Brandt-Straße, 52428 Jülich, Germany

**^‡^**These authors contributed equally to this work.

Corresponding author: Alexi K. Alekov, PhD, Medizinische Hochschule Hannover, OE4230, Carl-Neuberg-Str. 1, 30625 Hannover, Germany, Fax: ++49 511 532 9391,

Email: alexi.alekov@gmail.com

**List of Appendix Figures and Tables:**

**Appendix Figure S1 page 2**

**Appendix Figure S2 page 3**

**Appendix Figure S3 page 4**

**Appendix Figure S4 page 5**

**Appendix Figure S5 page 6**

**Appendix Figure S6 page 7**

**Appendix Figure S7 page 8**

**Appendix Table S1 page 9**

**Appendix Table S2 page 9**

**Appendix Table S3 page 10**

**Appendix References page 10**


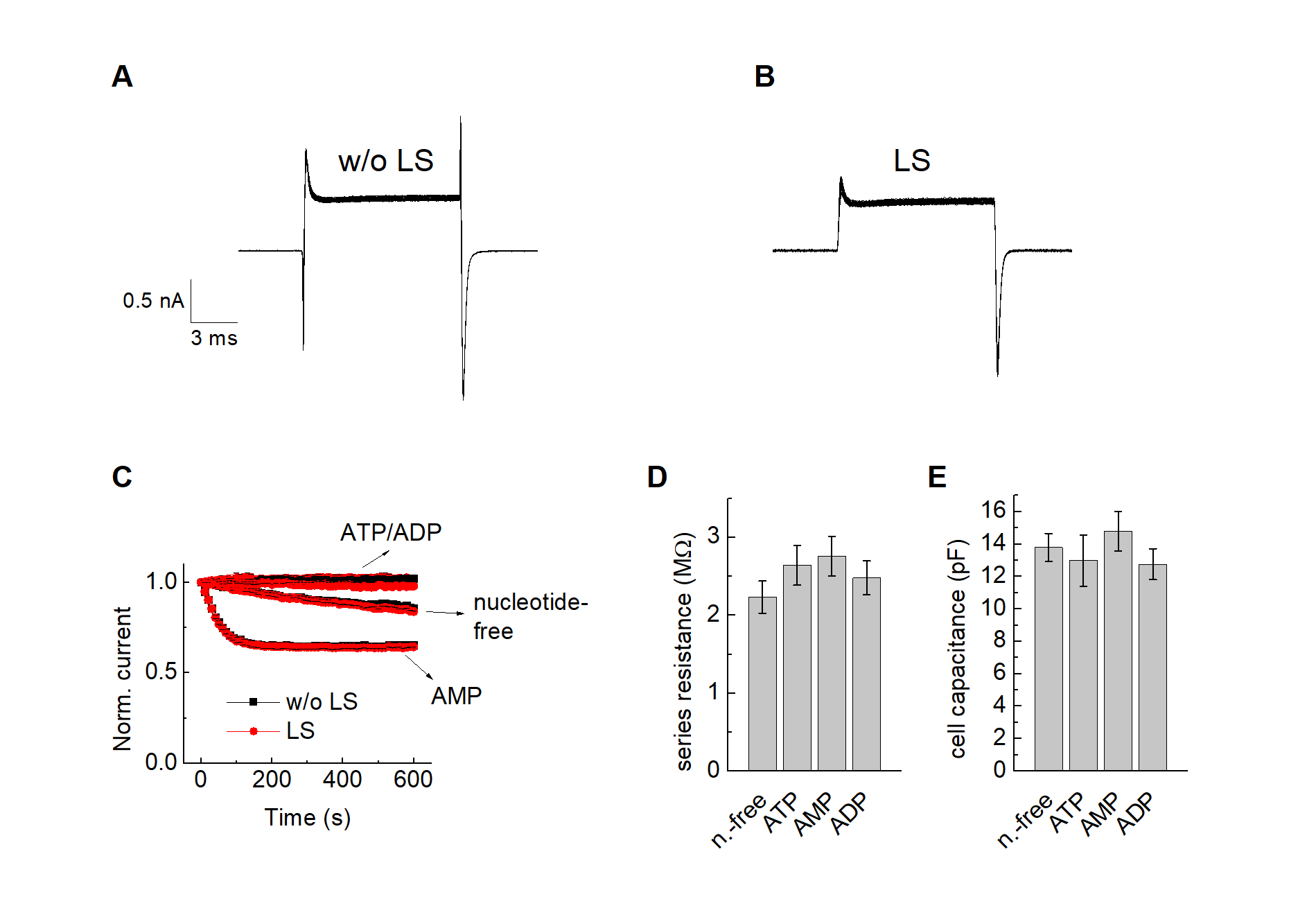


**Appendix Figure S1. Parameters of the experimental ClC-3 data set presented in Figure 1, main text. (A), (B)** Representative current traces from a cell expressing ClC-3 recorded without (A) and with (B) leak subtraction (same recording, displayed in two different modes). **(C)** Effects of adenine nucleotides or their washout on ClC-3 ion transport (n=5 in each data set, total 20). The current amplitudes were measured at the end of the test pulses using data as shown in (A, B), and normalized to the initial current amplitudes obtained after establishing the whole-cell configuration **(D), (E)** Series resistances and cell capacitances in the ClC-3 experiments shown in (C) as measures of the patch pipette diameters and cell sizes, respectively (“n.-free” indicates nucleotide-free measurements, n=5 in each data set, total 20 per pannel.


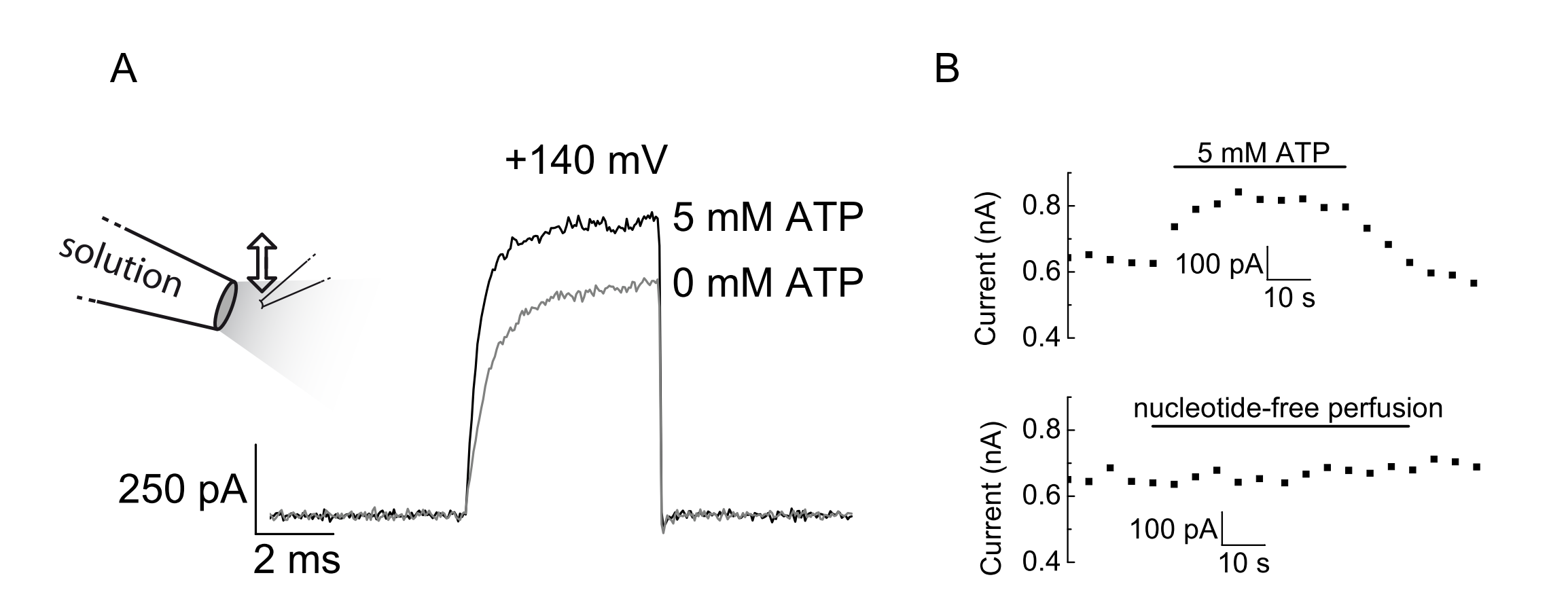


**Appendix Figure S2. Testing the reversibility of the ATP-induced increase of ionic ClC-5 transport.** (**A**) Left, scheme of the experimental perfusion setup. An inside-out patch pulled from a HEK293T cell expressing ClC-5 was moved through a local solution stream created by a large perfusion micropipette filled with ATP-containing pipette solution. In the beginning, the cytosolic side of the patch (and the ClC-5 C-terminus) is exposed to nucleotide-free pipette solution. Moving the patch in the perfusion stream exposes the cytosolic side of the patch to ATP-containing pipette solution. Right, a representative recording from an excised inside-out patch subjected to sequential cytosolic perfusion with pipette solution containing 5 mM ATP or nucleotide-free pipette solution. SCN^-^-based bath solution (filled in the patch pipette) was used in these experiments to increase the ClC-5 current amplitude. Currents were elicited by voltage jumps to +140 mV from a holding potential of 0 mV. The ATP-induced current increase was 1.37±0.3, n=4 (MW±SEM). Please note that SCN^-^ partially uncouples ClC transport resulting in slippage mode channel-like conductance that is not obligatory coupled to the antiport of protons (Accardi *et al*, 2006; Nguitragool & Miller, 2006; Walden *et al*, 2007; Zdebik *et al*, 2008; Alekov & Fahlke, 2009; Grieschat & Alekov, 2012). The effects are, therefore, not directly comparable to the effects in Cl^-^-based solutions. (**B**) Upper panel, steady-state current amplitude of the ClC-5 current measured in the same inside-out patch that is depicted in (A). Lower panel: control measurement of the same patch perfused with nucleotide-free pipette solution.

A B C


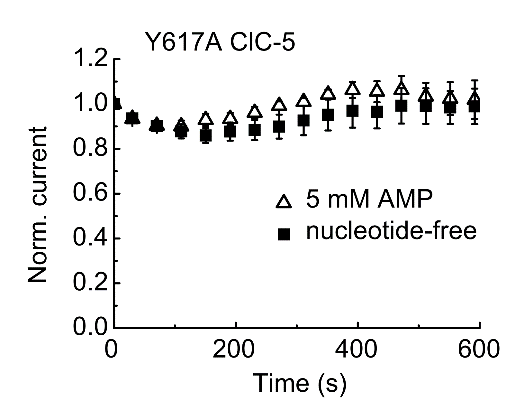

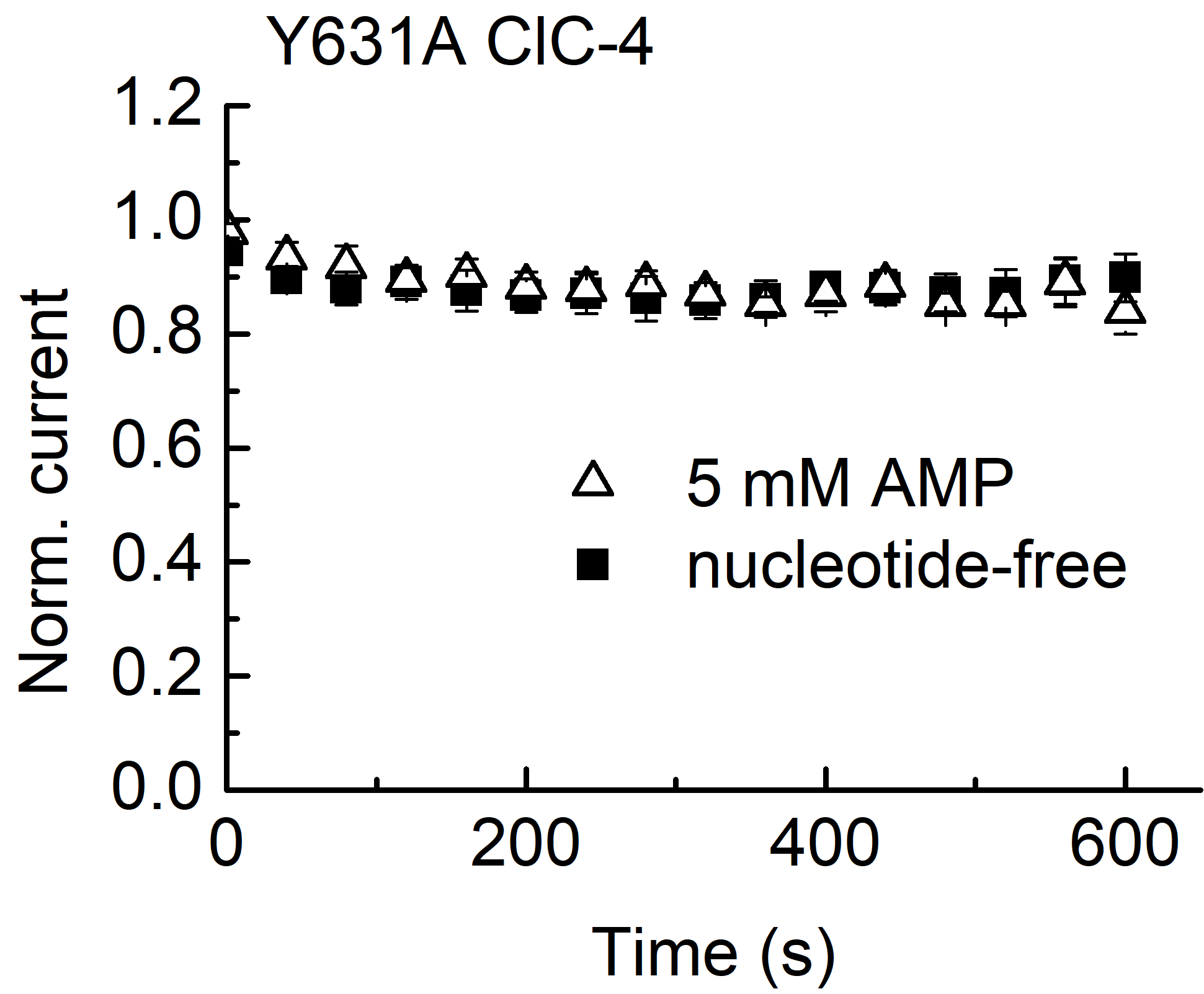


**Appendix Figure S3. Mutant Y617A and Y631A abolish the adenine nucleotide regulation of ClC-5 and ClC-4, respectively. (A)** Alignment showing the sequence of ClC-3, ClC-4, and ClC-5 around the nucleotide binding site responsible for the effects on CLC transport. **(B)** Normalized ClC-5 ion current amplitudes at +140 mV measured in transfected HEK293T cells after establishing whole-cell configuration with nucleotide-free internal solution or with 5 mM AMP added to the internal solution. Error bars represent SEM (n=5-6). **(C)** Normalized ClC-5 ion current amplitudes at +140 mV measured in transfected HEK293T cells after establishing whole-cell configuration with nucleotide-free internal solution or with 5 mM AMP added to the internal solution. Error bars represent SEM (n=6 and 4 for the nucleotide-free and AMP data, respectively).

**
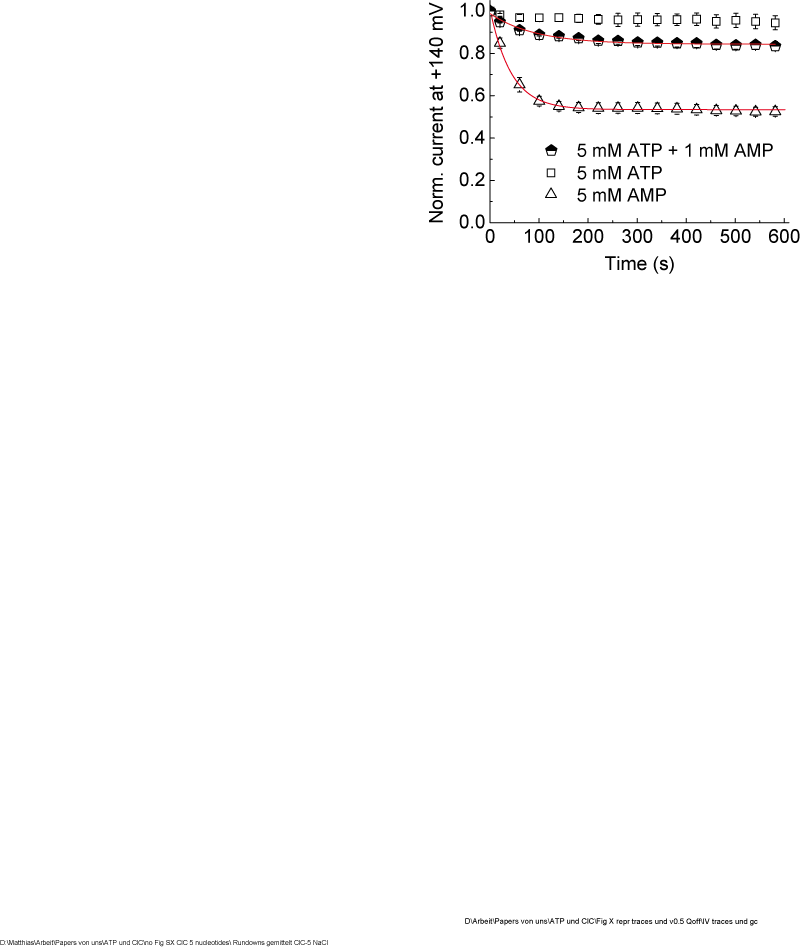
**

**Appendix Figure S4. Rundown of ClC-5 transport measured with 1 mM AMP and 1 mM ATP added together to the pipette solution.** ClC-5 currents were measured at +140 mV in HEK293T cells expressing ClC-5 after establishing whole-cell configuration with a pipette solution containing 5 mM ATP and 1 mM AMP (n=6). For comparison, the data from Fig. 1C obtained with 5 mM ATP or 5 mM AMP in the pipette are also depicted. Error bars represent SEM, red lines indicate monoexponential fits to the data. For the AMP/ATP mixture, the time constant and current decline after 600s were 93.1±23.9 s (SEM) and to 83.2±2.3% (SEM), respectively.


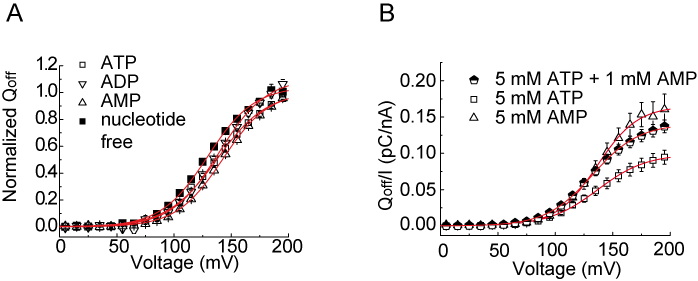


**Appendix Figure S5. Off-gating charge-voltage analysis of WT ClC-5 with different adenine nucleotides added to the pipet solution. (A)** Normalized off-gating **c**harge-voltage relationships of WT ClC-5 elicited by pulses from -115 mV to +195 mV (depicted is only the dynamic range) in presence or absence of adenine nucleotides in the pipette. The same data but normalized to the ionic current at +165 mV are depicted in Fig. 2B, (n=7-12). Red lines represent Boltzmann fits to the data. **(B)** Off-gating **c**harge-voltage relationships of WT ClC-5 elicited by pulses from -115 mV to +195 mV and normalized to the ionic current at +165 mV (depicted is only the dynamic range) in presence of 5 mM ATP and 1 mM AMP in the patch pipette. Red lines indicate Boltzmann fits to the data; the mean half-maximal voltage of activation for the ATP+AMP combination was 132.5±0.8 mV (n=6, MW±SEM). For comparison, the data from Figure 2B obtained with 5 mM internal ATP or internal 5 mM AMP are also depicted, parameters for these measurements are summarized in Appendix Table S1. All error bars indicate SEM.


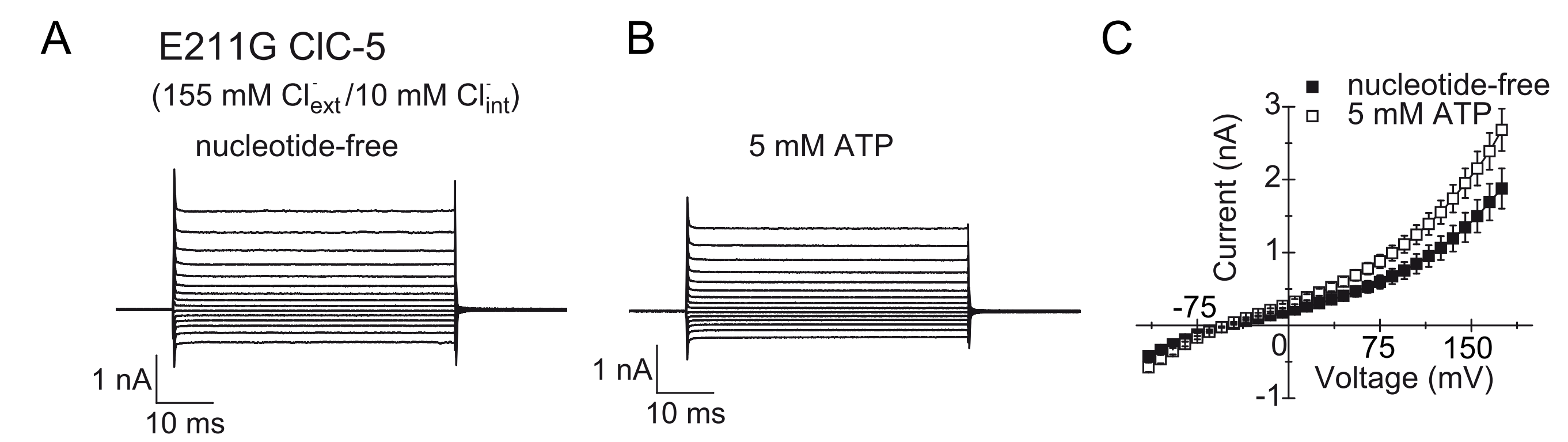


**Appendix Figure S6. Whole cell currents of the ungated mutant E211G ClC-5.** (**A, B)** Representative recordings of cells expressing E211G ClC-5 with nucleotide-free internal solution (A) or with 5 mM ATP in the pipette solution (B) upon 50-ms voltage pulses from -115 mV to +195 mV. P/4 leak subtraction was not applied. The internal Cl^-^ concentration was reduced to shift the ion current reversal potential and to ensure that the unspecific leak conductance contribution is negligible. (**C**) Mean steady-state current-voltage relationships of measurements with (n=5) or without (n=4) ATP added to the internal patch solution. Error bars show SEM.


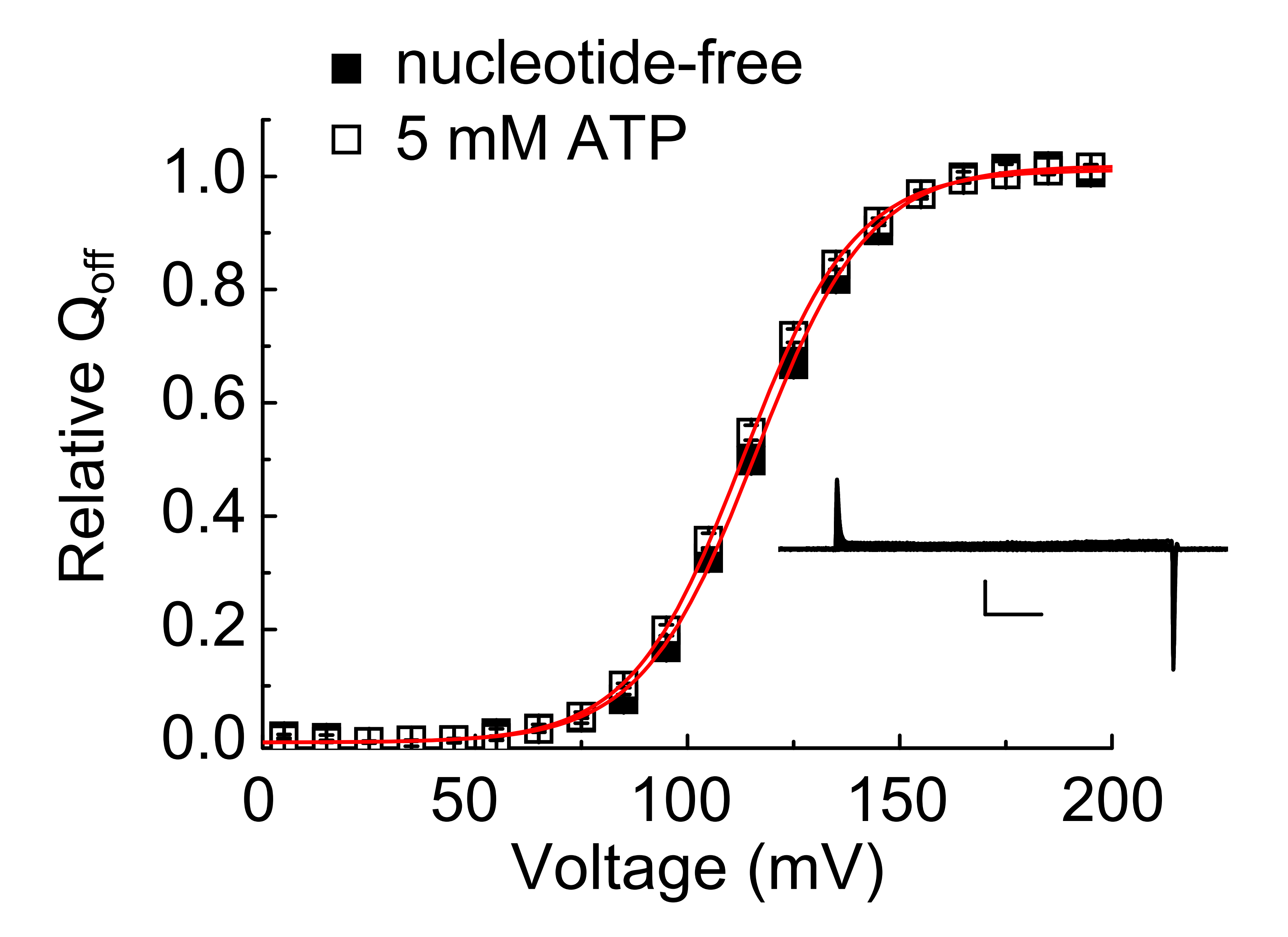


**Appendix Figure S7. Effects of ATP on the voltage dependence of mutant E268Q ClC-5.** Normalized off-gating **c**harge-voltage relationships calculated using gating currents of E268Q ClC-5 elicited by 50-ms pulses and measured with or with and without 5 mM ATP in the pipette solution. Red lines indicate Boltzmann fits to the data. Error bars represent SEM, n=5 in each data set. The inset shows a representative whole-cell recording of E268Q ClC-5. The mutant blocks the internal proton access, abolishes ion transport and increases the on- and off-gating currents (Grieschat & Alekov, 2012). Scale bar indicates 10 ms and 1 nA in the X- and Y-axis, respectively. The fit parameters are summarized in Appendix Table S3.

**Appendix Table S1. Boltzmann fit parameters describing the charge-voltage relationships of ClC-5 measured with a nucleotide-free pipette solution or with pipette solutions containing different adenine nucleotides (MW±SEM).**

|  | **5 mM ATP** | **5 mM ADP** | **5 mM AMP** | **nucleotide-free** |
| --- | --- | --- | --- | --- |
| midpoint of activation *V_0.5_* (mV) | 138.2 ± 2.2** | 137.8 ± 1.9** | 143.4 ± 1.7*** | 128.0 ± 1.7 |
| *unitary gating charge z* | 1.18 ± 0.03 | 1.28 ± 0.06 | 1.27 ± 0.03 | 1.30 ± 0.01 |
| *n* | 12 | 7 | 15 | 9 |

Unitary gating charge is presented as apparent number of elementary charges *e_0_* per transporter. Fit results refer to the data presented in Figure 2B. Asterisks display significant differences towards the nucleotide-free condition (two-sample t-test, *, p<0.05; **, p<0.01; ***, p<0.001).

**Appendix Table S2. Boltzmann fit parameters describing the charge-voltage relationships of ClC-3 measured with a nucleotide-free pipette solution or with pipette solutions containing different adenine nucleotides (MW±SEM).**

|  | **5 mM ATP** | **5 mM ADP** | **5 mM AMP** | **nucleotide-free** |
| --- | --- | --- | --- | --- |
| midpoint of activation *V_0.5_* (mV) | 72.0 ± 0.9** | 75 ± 1* | 93.6 ± 0.8* | 83.4 ± 0.8 |
| *unitary gating charge z* | 1.5 ± 0.1 | 1.5 ± 0.1 | 1.7 ± 0.2 | 1.30 ± 0.1 |
| 2^nd^ midpoint of activation *V_0.5_* (mV) | - | - | ~600 | - |
| *2^nd^ unitary gating charge z* | - | - | ~0.3 | - |
| *n* | 4 | 4 | 5 | 4 |

Unitary gating charge is presented as apparent number of elementary charges *e_0_* per transporter. Fit results refer to the data presented in Figure 2C. For the AMP dataset, a fit with the sum of two standard Boltzmann functions (see Eq. 1 in Methods) was required to satisfactory describe the data. As the saturating part of the activation curve was not reached in the investigated voltage range, the parameters of the second Boltzmann function are not well defined (two-sample t-test with nucleotide-free conditions as a reference, *, p<0.05; **, p<0.01; ***, p<0.001).

**Appendix Table S3. Boltzmann fit parameters describing the charge-voltage relationships of mutant E268Q ClC-5 with or without ATP added to the pipette solution (MW±SEM).**

|  | nucleotide-free | 5 mM ATP |
| --- | --- | --- |
| midpoint of activation *V_0.5_* (mV) | 116.2 ± 1.6 | 113.2 ± 0.7 |
| *unitary gating charge z* | 1.89 ±0.07 | 1.94 ± 0.01* |
| *n* | 5 | 5 |

Unitary gating charge is presented as apparent number of elementary charges *e_0_* per transporter. Fit results refer to the recordings in Appendix Figure S7. Asterisks indicate statistical significance evaluated using a two-sample t-test with nucleotide-free conditions as a reference, (* - p<0.05).

**Appendix References**

Accardi A, Lobet S, Williams C, Miller C & Dutzler R (2006) Synergism Between Halide Binding and Proton Transport in a CLC-type Exchanger. *J. Mol. Biol.* **362:** 691–699

Alekov AK & Fahlke Ch (2009) Channel-like slippage modes in the human anion/proton exchanger ClC-4. *J. Gen. Physiol.* **133:** 485–496

Grieschat M & Alekov AK (2012) Glutamate 268 Regulates Transport Probability of the Anion/Proton Exchanger ClC-5. *J. Biol. Chem.* **287:** 8101–8109

Nguitragool W & Miller C (2006) Uncoupling of a CLC Cl−/H+ Exchange Transporter by Polyatomic Anions. *J. Mol. Biol.* **362:** 682–690

Walden M, Accardi A, Wu F, Xu C, Williams C & Miller C (2007) Uncoupling and turnover in a Cl-/H+ exchange transporter. *J. Gen. Physiol.* **129:** 317

Zdebik AA, Zifarelli G, Bergsdorf E-Y, Soliani P, Scheel O, Jentsch TJ & Pusch M (2008) Determinants of Anion-Proton Coupling in Mammalian Endosomal CLC Proteins. *J. Biol. Chem.* **283:** 4219–4227
